# Breeding Dispersal by Birds in a Dynamic Urban Ecosystem

**DOI:** 10.1371/journal.pone.0167829

**Published:** 2016-12-28

**Authors:** John M. Marzluff, Jack H. DeLap, M. David Oleyar, Kara A. Whittaker, Beth Gardner

**Affiliations:** School of Environmental and Forest Sciences, University of Washington, Seattle, Washington, United States of America; Liverpool John Moores University, UNITED KINGDOM

## Abstract

Changes in land cover during urbanization profoundly affect the diversity of bird communities, but the demographic mechanisms affecting diversity are poorly known. We advance such understanding by documenting how urbanization influences breeding dispersal—the annual movement of territorial adults—of six songbird species in the Seattle, WA, USA metropolitan area. We color-banded adults and mapped the centers of their annual breeding activities from 2000–2010 to obtain 504 consecutive movements by 337 adults. By comparing movements, annual reproduction, and mate fidelity among 10 developed, 5 reserved, and 11 changing (areas cleared and developed during our study) landscapes, we determined that adaptive breeding dispersal of sensitive forest species (Swainson’s Thrush and Pacific wren), which involves shifting territory and mate after reproductive failure, was constrained by development. In changing lands, sensitive forest specialists dispersed from active development to nearby forested areas, but in so doing suffered low annual reproduction. Species tolerant of suburban lands (song sparrow, spotted towhee, dark-eyed junco, and Bewick’s wren) dispersed adaptively in changing landscapes. Site fidelity ranged from 0% (Pacific wren in changing landscape) to 83% (Bewick’s wren in forest reserve). Mate fidelity ranged from 25% (dark-eyed junco) to 100% (Bewick’s wren). Variation in fidelity to mate and territory was consistent with theories positing an influence of territory quality, asynchronous return from migration, prior productivity, and reproductive benefits of retaining a familiar territory. Costly breeding dispersal, as well as reduced reproductive success and lowered survival cause some birds to decline in the face of urbanization. In contrast, the ability of species that utilize edges and early successional habitats to breed successfully, disperse to improve reproductive success after failure, and survive throughout the urban ecosystem enables them to maintain or increase population size.

## Introduction

In an increasingly urban world [1], scientists are only beginning to quantify basic ecological processes that characterize the ecosystems humans call home [2, 3]. Unique biogeochemical cycles [4], energy flows [5], and trophic relationships [6, 7] interact with altered disturbance regimes and human preferences to create a unique ecological stage in the city and its surrounding suburbs and exurbs [8–11]. Here, general patterns of biological diversity are well known [12–16], but the adjustments of population processes leading to these patterns are understudied [17]. The species that come to dominate urban systems are behaviorally flexible [18], which may enable them to reproduce [19–22], survive [22, 23], disperse from natal areas [24–26], compete [27–29], avoid predation [7], and engage humans [30–32] more effectively than less flexible species that are extirpated.

Breeding dispersal—the annual shift in an adults’ center of reproductive activity [33]—remains one of the least understood yet fundamental processes by which animal populations adapt to their environment [34]. Breeding dispersal reflects not only an individual’s decision to remain faithful to a site, but also often its decision to remain faithful to a mate. Breeding dispersal in birds is generally of shorter distance than natal dispersal, often more extensive by females than males, and motivated by differential costs of movement and benefits of retaining one’s mate or territory [33, 35]. Costs and benefits are affected by a rich mix of individual and environmental characteristics, including population density, territory quality, pair compatibility, success and quality of neighbors, actions of predators and parasites, and an individual’s sex, social status, age, and previous experience [34, 36–38]. In urban environments breeding dispersal is rarely studied [39] and may be influenced by the above factors as well as the actions of humans that benefit or challenge birds [40].

Our objective is to describe breeding dispersal and mate fidelity in a variety of songbirds that inhabit the rapidly urbanizing forests surrounding the city of Seattle, WA, USA. We organize our exploration around three research questions: 1) Does a species’ life history influence site fidelity and mate fidelity? 2) Does annual productivity, mate fidelity, or landscape conversion influence annual movement of breeders and likelihood of divorce? 3) Does movement improve reproduction or enable dispersers to settle in appropriate habitat? In so doing, we test several predictions (Table 1) across multiple species and within a unique setting that includes forest reserves and existing developments where human actions were relatively constant during our study, as well as within forests that were actively developed into suburbs during this time [22]. Investigating the first question enables us to test the hypothesis that asynchronous return to territory in species that migrate may reduce site fidelity (Table 1 1A) and mate fidelity (Table 1 1B) because early arriving males hedge their bets [41] or late arriving females select mates based on territory quality rather than prior breeding experience [42]. Exploring the second question allows us to assess two more hypotheses. First, we expect reproductive failures to stimulate breeding dispersal (Table 1, 2A) and reduce mate fidelity (Table 1, 2E) [43, 44]. Additionally, we expect breeding dispersal distance to increase (Table 1, 2B, 2C and 2F) and mate fidelity to decrease where habitats of varying quality exist near one-another because birds with poor quality territories will move to attain high quality territories (Table 1, 2D and 2E) [34, 45, 46]. Finally, the third question motivates a test of the hypothesis that following failure, increased number or quality of young are produced by individuals that disperse farthest or abandon mates to pursue better options, however this presumed benefit is inconsistently realized (Table 1, 3A–3F) [38, 47]. We expect the decisions of all species to be mediated by the costs and benefits of fidelity, as hypothesized above, however where subdivisions are being built the adaptive decisions of sensitive species may be constrained by the rapid loss of their preferred habitats. In environments of fluctuating resource availability, such as this, breeding dispersal may be exaggerated and not confined to individuals of low quality [48].

**Table 1:** Research questions, predictions from hypotheses, and statistical approaches to testing each.

Research Question and Prediction from Hypothesis	Response Variable	Explanatory Variables (bold)	Statistical Procedure
1) Does a species’ life history influence mate and site fidelity?			
A. Less site fidelity in migrants than non-migrants	Number of pairs	**Migrant status** (long- and short-distance versus resident) and **Site Fidelity**	Fisher’s Exact Test
B. Less mate fidelity in migrants than non-migrants	Number of pairs	**Migrant status** of 3 well-sampled exploiter/adapters (1 short-distance migrant versus 2 resident species) and **Mate Fidelity**	Fisher’s Exact Test
2) Does annual productivity, mate fidelity, or landscape conversion influence annual movement of breeders and likelihood of divorce?			
A. Greater annual movement by failed breeders than successful breeders	Annual distance moved between territory centers	**Fledging success in previous year** (yes or no), **Landscape, Guild, interaction of Landscape and Fledging success**	Generalized Linear Mixed Model—with site included as a random effect (see S3 Table for full results)
B. Greater annual movement in changing lands than stable lands	Annual distance moved between territory centers	**Landscape, Guild, interaction of Landscape and Guild**	Generalized Linear Mixed Model—with site included as a random effect (see S6 Table for full results)
C. Greater annual movement by avoiders than adapters/exploiters	Annual distance moved between territory centers	**Landscape, Guild, interaction of Landscape and Guild**	Generalized Linear Mixed Model—with site included as a random effect (see S6 Table for full results)
D. Greater annual movement by divorcees than those retaining mate	Annual distance moved between territory centers	**Landscape, Status of pair bond** of 3 well-sampled exploiter/adapters (intact, broken due to divorce, broken due to death)	Generalized Linear Mixed Model—with site included as a random effect (see S4 Table for full results)
E. Greater frequency of divorce by failed breeders than successful breeders	Number of pairs of 3 well-sampled exploiter/adapters	**Fledging success in previous year** (yes or no) and **Divorce versus retain mate**	Fisher’s Exact Test
F. Greater annual movement from territories experiencing local habit modification greater than those not modified	In Changing Landscapes only: Annual distance moved between territory centers	**Pixels of territory converted** from forest, to built land, or to other land cover	Correlation
3) Does movement improve reproduction or enable dispersers to settle in appropriate habitat?			
A. Greater fledging success after moving in response to failure in reserves than in changing lands	Number of pairs of avoiders	**Landscape** (changing or reserve) and **Fledging success in year after movement** (yes or no)	Fisher’s Exact Test
B. Improved fledging success after movement by failed breeders in reserves but not changing lands	Number of pairs of avoiders	**Change in fledging success** (worse versus same or better) and **Landscape** (changing or reserve)	Binomial Regression
C. Reduced fledging success after failure if site faithful, but increased success following dispersal	Number of pairs of exploiters/adapters	**Change in fledging success** (success after failure or failure after success) and **Site fidelity** (yes or no)	Fisher’s Exact Test
D. Increased forest cover in territory of avoiders	Amount of forest within 100m of territory center	**Territory location** (abandoned versus obtained), **Guild**	ANOVA
E. Distance moved increases territory quality for avoiders	Annual distance moved between territory centers	**Pixels of forest cover gained**, **Prior success at fledging young** (Yes or No)	Generalized Linear Mixed Model—with site included as a random effect (see S7 Table for full results)
F. Distance moved increases territory quality for exploiters/adapters	Annual distance moved between territory centers	**Pixels of non-forested cover gained**, **Prior success at fledging young** (Yes or No), **Mate retention** (Yes or No)	Generalized Linear Mixed Model—with site included as a random effect (see S8 Table for full results)

We studied six species of socially monogamous, ground- and shrub-nesting birds (Bewick’s wren, *Thryomanes bewickii*; dark-eyed junco, *Junco hyemalis*; Pacific wren, *Troglodytes pacificus;* song sparrow, *Melospiza melodia*; spotted towhee, *Pipilo maculatus*; and Swainson’s thrush, *Catharus ustulatus*) that differ in response to urbanization and migratory behavior. The Swainson’s thrush is a neotropical migrant and the dark-eyed junco is a local migrant, but the other species are resident on our study sites throughout the year [49–54]. We categorize two species (Pacific wren and Swainson’s thrush) as ‘avoiders’ of urban lands (*sensu* [12, 55]) because they decline in abundance as urbanization proceeds and are generally rare in suburban and urban landscapes [22, 29]. We consider the other four species ‘adapters’ and ‘exploiters’ (as defined by [12] or as considered to be ‘dependents, exploiters, and tolerants’ by [55]) because their abundance remains stable (song sparrow, spotted towhee) or increases (dark-eyed junco, Bewick’s wren) as lands are developed and they are abundant in suburban and urban landscapes [22, 29]. We did not explicitly account for phylogenetic relationships in our study, but note that closely related species occur across our sampling design, for example we include migratory (dark-eyed junco) and resident (song sparrow, spotted towhee) species from the same family (Emberizidae), as well as avoiders (Pacific wren) and adapters (Bewick’s wren) from the same family (Troglodytidae). Territory fidelity is unreported in spotted towhees [54], but known to be high in the other species in non-urban areas [50–53]. Much less is known about mate fidelity, though it is relatively high in both song sparrows [49] and dark-eyed juncos [51] inhabiting undisturbed islands and forests, respectively.

## Materials and Methods

### Permits and Ethics

All research reported was approved by the IACUC committee at the University of Washington (protocol 3077–01) and permitted by Washington Dept. of Fish and Wildlife (research permit MARZLUFF 15–164) and the US Federal Bird Banding Lab (banding permit 22489).

### Study Sites

In 1998 and 1999 we selected 26 long-term study sites arranged in a quasi-experimental design across the rapidly developing forests east of Seattle, WA, USA (for map see [22]). The sites are characterized by second-growth, 70-100-year-old coniferous forest below 500 m in elevation and include five ‘Reserves’ (75–500 ha recreation, natural area, and watershed conservation forests), 10 single-family-home neighborhoods developed 20–50 years ago (‘Developed’), and 11 ‘Changing’ sites (the study area is detailed in [56–58]. ‘Changing’ sites provide insights into the bird community before, during, and after creation of single-family-home neighborhoods [22, 59]. We monitored birds in these changing landscapes from the onset of development, typically having 1–2 years of research completed before extensive clearing, roading, and building occurred. This design enables insights into how development affects the movement of adult birds by allowing us to make comparisons through time at these sites with reference to a separate set of nearby reserved forests and developments that experience the same climatic conditions and draw from the same pool of birds, but do not experience changes in forest or built land cover. Reserves and developments were selected from a larger random sample (described in [56, 60]) to be spatially interspersed with changing sites. Changing sites were not randomly selected, but rather we learned opportunistically about pending developments and selected those that were accessible and planned for imminent construction.

### Field Efforts

We spent 120–240 h per site per year from 2000–2010. We spent the majority of this time between dawn and 15:00 (PST) in the forested remnants in each site mist-netting and color banding birds (4–10, 6-hr sessions per site) and recording the locations of breeding and territorial activities, as well as the occurrence of previously marked birds (16–32, 5-hr sessions per site) using GPS and field maps of each study area. We netted 2396 adult (after hatch year) birds using both passive capture and attraction to playback of territorial songs. We uniquely color-banded and noted plumage, condition of brood patch and cloacal protuberance of each captured bird to determine age and gender [61]. During weekly visits to each site, we searched for nests and ‘spot mapped’ the activity of adults within each territory [62]. From 2002–2010, we mapped 6363 territories (noting at least weekly for a minimum of 8 weeks the location of breeding pairs) and while mapping found 418 nests. As an index of territory size, we estimated the maximum radius across the minimum convex polygon surrounding all locations of marked pairs [29]. We determined annual productivity within these territories by monitoring parental behavior from the onset of breeding activity throughout the spring and summer until the end of the final nesting attempt, mapping the locations of these behaviors, and assessing pairing status, the number of successful breeding attempts, fledglings per attempt, and total annual production of fledglings [63–65]. Here we categorize pairs as ‘successful’ if they fledged one or more broods within a year and ‘unsuccessful’ if they failed to fledge young. We assessed different total areas among sites (mean = 6.1±0.9 ha) because of differences in forested area, bird density, and accessibility, but our efforts resulted in similar assessments across sites (i.e., delineation of all territories in forest areas).

### Determination of Dispersal Distance

The basic observation that we analyzed in this paper is the location of a color-banded, territorial adult in two consecutive years. From 2002–2010 we obtained 504 consecutive locations on 337 adults (hereafter ‘movements,’192 in changing, 67 in reserve, and 78 in developed sites). We measured the distance between successive year’s territory centroids (the nest site, when known, or the central point with greatest activity observed during spot mapping, when nest was not located; as in [34] in m, using a portable GPS in the field or after assessing locations of activity on high resolution satellite imagery and field maps generated by geographic information systems at the end of the field season. We define breeding site fidelity for each species as movements less than the species-specific average maximum radius of territories we measured in each study landscape. We were unable to ascertain the productivity of all pairs every year; therefore, our sample of movements with known history is a subset of these 504 moves. We determined the annual productivity in the first year of 371 consecutive pairs of locations (prior to movement) made by 285 birds and the annual productivity in the second year of 327 pairs of locations (after movement) made by 226 birds. In 157 cases we determined if the individual remained paired with its prior mate after the movement.

### Quantifying Change in Landscape Composition

To relate movements to changes in the composition of the landscape we gathered all available USGS, IKONOS, and GOOGLE EARTH orthoimagery of the study area into ArcGIS 10.0 [66]. We implemented a two-part landscape classification process using five categories. The first four cover types were modified from [12]: *Forest*, *Built* (impervious surfaces such as buildings, roads, sidewalks), *Bareground-grass* (bare ground, grass/lawn), *Shrub* (woody shrub or early seral/regenerating forest), with the addition of a fifth cover type open *Water* (sloughs and naturally occurring or engineered-retention ponds). We first subjected orthoimages to Object Based Image Analysis (OBIA; *sensu* [67]). OBIA is multi-step process using SPRING 5.2 software [68], that first implements algorithms designed to discriminate among object boundaries by aggregating pixels into like entities prior to reliance on spectral reflectance. Next, OBIA classifies imagery by iteratively identifying relevant multi-pixel ‘objects’ through ‘training data,’ for each class. Because orthoimagery available during this time period included black and white (2-spectrum), 3-spectrum color, and 4-spectrum color (3 visible spectrums plus infrared) we further enhanced image interpretation through visual inspection, including comparison of classified landscapes to historical imagery online (Google Earth, 2014), and editing classified landscapes through extensive free-hand digitizing of mis-classified areas. Because selecting and implementing a reliable methodology for assessing classification accuracy of remotely sensed imagery from such varied sources is extremely challenging [69], we focused on the elimination of inter-operator bias [70] by constraining the process to one individual.

**Fig 1:**
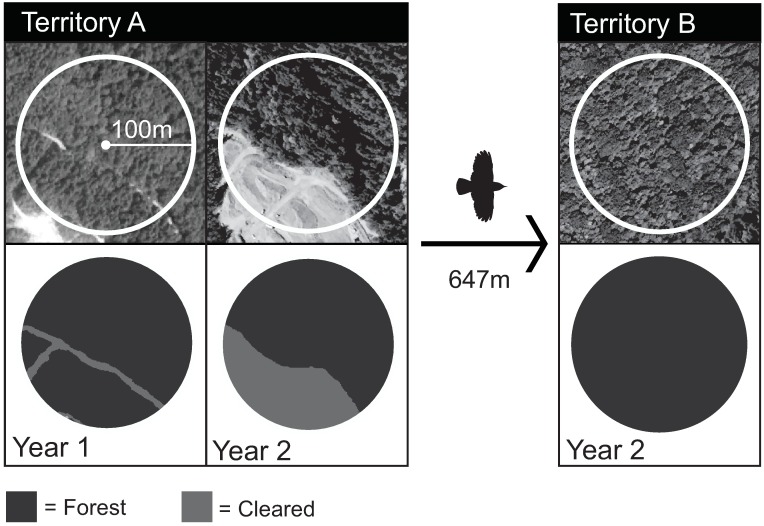
Classification of satellite images (top row) into suburban vegetation classes (bottom row) at original breeding site during year of occupancy (Territory A, Year 1), original breeding site during year it was abandoned (Territory A, Year 2), and new breeding site during year of occupancy (Territory B, Year 2). In this example of a Pacific wren, the breeding male moved 647m from Territory A to Territory B in association with clearing of a portion of his former territory for construction of a new subdivision.

We calculated the composition of the landscape (proportion of each category within a circle of radius 100m emanating from the territory centroid) before and after 284 movements in changing study sites (all with adequate imagery; 22 movements of avoiders and 262 movements of adapters/exploiters). From these consecutive, annual assessments, we measured: a) the difference in landscape composition at the territory that was abandoned, which equals the amount of forested or developed land in year 1 minus those amounts at the same place in year 2 (Territory A: Year 1-Year 2, Fig 1); and b) the change in landscape composition that was obtained by moving, which equals the difference in the amount of forested or developed land in the territories in year 2 (Territory A Year 2 –Territory B Year 2, Fig 1b).

### Statistical Approach

We conducted all statistical analyses using SPSS (v.19; [71]) and R (v 3.2.5; [72]). When comparing movements between guilds or landscapes we considered the average movements by an individual bird as the experimental unit. Averaging enables each individual bird to be included in these broad analyses regardless of our knowledge of individual histories. However, when relating movement to annual productivity, fidelity to mate, and land cover in the territory, we followed [34] and considered an individual’s annual movement to be the experimental unit because each movement was associated with a unique history (preceded by a unique reproductive event that was often conducted with a different partner in a territory that was in a unique location surrounded by different neighbors and where human action frequently changed the local vegetation). We used standard parametric or exact tests to investigate the relationship between variables other than dispersal distance. We used generalized linear mixed models (GLMMs) to understand how dispersal distance was influenced by four categorical factors: 1) annual production of fledglings; 2) fidelity to mate (resulting movements where pair remained intact or where new bonds formed after death or divorce of partner); 3) landscape (changing, developed, and reserve sites); 4) guild (avoider versus adapter/exploiter), and two continuous covariates: 1) change in local landscape within territory that was abandoned (number of pixels of each land cover class gained or lost), and 2) change in local landscape within territory that was obtained (pixels gained or lost by cover class). Because the distribution of dispersal distances has positive support and a long right tail, we used the gamma distribution with a log link in all GLMMs. We standardized the continuous covariates and included site as a random effect in the models to account for potential variation occurring at the site level. We also used generalized linear models to test the influence of landscape on the likelihood of fledging young before and after a movement. For our relatively small sample of avoiders, we quantified change in fledging success before and after movement as the ‘same or better’ versus ‘worse’ using binominal regression with a probit link. We report raw factor means and standard errors (and medians for distance moved (S1 Table), and include test statistics to indicate significance based on the corresponding model. While much of our approach is descriptive and exploratory, we used the aforementioned tests to appraise predictions from our four hypotheses (Table 1).

We follow Fisher’s [73] (see also [74]) approach to statistical inference and use p-values to screen for potentially real or useful associations that have merit for future investigation. We report p-values and interpret those <0.05 as providing evidence of an effect that should be confirmed with other studies. P-values between 0.05 and 0.20 provide evidence of an effect that should be tested with additional studies of improved design (e.g., increased replication). P-values >0.20 indicate that if there is an effect it is too small to detect with the current experimental design. In our analysis, we do use partially overlapping datasets; however because we test specific predictions from *a priori* hypotheses and offer a broad interpretation of the p-values, we did not employ any corrections for multiple comparisons [75].

## Results

### Does a Species’ Life History Influence Site Fidelity and Mate Fidelity?

**Fig 2:**
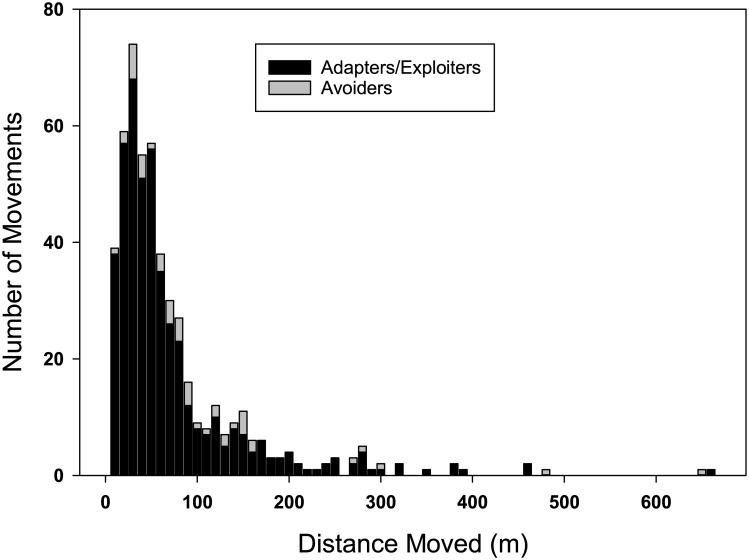
Breeding dispersal by songbirds in an urbanizing environment. The frequency of movements by distance category is separated by guild in this stacked bar figure (gray portion of bars is 47 distances moved by avoiders and black portion is 457 distances moved by adapters and exploiters).

Annual movements between the centers of a breeding songbird’s successive territories were slight (median = 44.4m) and their distribution was positively skewed with a sharp peak and long tail (Fig 2; skew = 3.26±0.11, kurtosis = 15.01±0.22, n = 504 moves by 337 adults). The smallest species by mass, the Pacific wren, which is a resident and an avoider exhibited the greatest annual movements while the medium-sized, resident Bewick’s wren moved least (median values: Pacific wren = 101.8m, n = 24 birds; Bewick’s wren = 32.6m, n = 9 birds). Species that migrate, including a locally migrant junco and long-distance migrant thrush dispersed greater distances (Swainson’s thrush = 68.4m, n = 17 birds; dark-eyed junco = 85.0m, n = 27 birds) than all resident species other than the Pacific wren (song sparrow = 39.3m, n = 162 birds; spotted towhee = 47.2m, n = 98 birds). The longest movement was of 653m by a male spotted towhee in a reserve.

Site fidelity varied among species (Table 2; X^2^_(5)_ = 24.3, P <0.0001). Territory size varied among species (F_5,1561_ = 14.5, P<0.001) and landscape (F_2, 1561_ = 4.7, P = 0.009), therefore we appraised site fidelity separately by species within landscapes (S2 Table). Swainson’s thrushes and Bewick’s wrens had the largest territories, while Pacific wrens and song sparrows had the smallest (Table 2). Appraising the distance moved with respect to the size of each species’ territories (Table 2), dark-eyed juncos and Pacific wrens exhibited the least site fidelity. Swainson’s thrushes also had low site fidelity. Song sparrows and spotted towhees showed intermediate levels of site fidelity at approximately 50%. Bewick’s wrens, which maintained the largest territories in our study, were most faithful to site. Bewick’s wrens only centered their activities beyond the average maximum territory radius 35.7% of the time (Table 2). Overall, the two migrant species were less faithful to their sites than were the four resident species (Fisher’s Exact test: P = 0.001).

**Table 2:** Territory size, site fidelity, apparent death of partner, and mate fidelity of songbirds spot-mapped in forested landscapes surrounding Seattle, WA, USA, from 2003–2010. Assessment of site fidelity was done within each landscape and summed here for an overall proportion because territory size varied among species and among landscapes (S1 Table). When a banded bird remated with a new partner and it’s mate from the prior year was not detected in the study area, we concluded the prior mate was apparently dead. Species abbreviated as follows, Bewick’s wren: BEWR; dark-eyed junco: DEJU; song sparrow: SOSP; spotted towhee: SPTO; Swainson’s thrush: SWTH; Pacific wren: PAWR.

SPECIES	Average Maximum Territory Radius (${\bar{x}}_{r}$) (n, SE)	Site Fidelity (% of movements < ${\bar{x}}_{r}$) (n moves)	Percentage of new pair bonds resulting from apparent death of one partner (n)	Mate Fidelity (% of resighted birds that remated with prior partner) (n)
**Guild: Adapter/Exploiter**				
**BEWR**	46.1m (151, 1.0)	64.3% (14)	50.0% (4)	100% (2)
**DEJU**	41.6m (205, 1.4)	16.1% (31)	46.7% (15)	25% (8)
**SOSP**	32.9m (369, 0.9)	49.8% (257)	50.6% (87)	74% (43)
**SPTO**	44.2m (360, 1.0)	49.0% (155)	56.5% (46)	70% (20)
**Guild: Avoider**				
**PAWR**	36.6m (268, 0.8)	18.5% (27)	100% (1)	NA
**SWTH**	49.6m (226, 1.1)	35.0% (20)	100% (4)	NA

Fidelity to mate was generally lower than fidelity to territory (Table 2). The majority of birds were mated with a new partner on the new territory gained from a movement (68.1% of 157 birds), mostly because of the apparent death of one partner. We observed 23 instances (21.5% of instances where both members of a pair were observed in subsequent years) of divorce in exploiters/adapters. Divorce was more frequent in the short-distance migrant junco (6/8 dark-eyed juncos) than in the resident species combined (10/42 song sparrows plus 6/20 spotted towhees; Fisher’s Exact Test P = 0.01). Divorce was not confirmed in any of the few subsequent bonds we observed of avoiders, Table 2).

### Does Annual Productivity, Mate Fidelity, or Landscape Conversion Influence Annual Movement of Breeders?

Annual reproductive failure preceded long movements, especially in changing landscapes. We documented 102 movements by birds after they failed to fledge young within a season and 269 movements after they succeeded to fledge at least one brood. Over all landscapes and guilds the distance moved following failure ($\bar{x}=74\pm 7.1\text{m}$) was not significantly different than movement following success ($\bar{x}=62\pm 4.6\text{m}$; P = 0.21). However, in changing landscapes where territory quality may be impacted by construction activities that modify the juxtaposition of high and low quality territories, we discovered that breeders moved significantly less after succeeding to fledge young ($\bar{x}=58.4\pm 4.72\text{m}$, n = 158 moves) than those failing to fledge young ($\bar{x}=78.9\pm 10.1\text{m}$, n = 52 moves; marginal effect of fledging success in changing landscapes P = 0.04; median values in S1 Table; full results in S3 Table).

Considering only well-sampled species (song sparrows, spotted towhees and dark-eyed juncos; Table 2) across all landscapes, the distance moved coincident with divorce ($\bar{x}=137.4\pm 28.4\text{m}$, n = 22) was double that moved in association with a partner’s apparent death ($\bar{x}=59.9\pm 7.2\text{m}$, n = 78) or when the partner was retained ($\bar{x}=60.7\pm 13.4\text{m}$, n = 48; P<0.001; median values in S1 Table; full results in S4 Table). Regardless of gender, dispersal distances of these species were largest following divorce (n = 12 females: $\bar{x}=139.3\pm 34.2\text{m}$; n = 11 males $\bar{x}=127.4\pm 45.2\text{m}$; S5 Table). Pairs that failed to fledge young were no more likely to divorce than were pairs that successfully fledged young (Fisher’s Exact Test P_(1-tailed)_ = 0.42).

**Fig 3:**
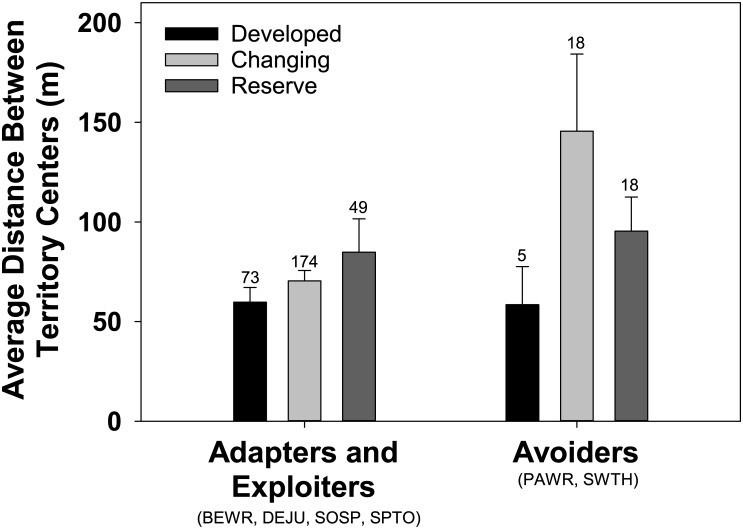
Average (+1SE) breeding dispersal distance by guilds in each of three landscapes. Sample size above error bars.

Ongoing conversion of forests to housing developments within changing landscapes affected the breeding dispersal of avoiders differently than that of exploiters/adapters. Each species responded in one of three ways to different landscapes (S1 Fig). Exploiters/adapters, such as song sparrows, spotted towhees, and Bewick’s wrens moved farthest in reserves or developments, but avoiders, such as Swainson’s thrushes and Pacific wrens, moved farthest in changing landscapes. As a result, there was a moderately significant difference in a guild’s average movement in changing landscapes (marginal effect of guild in changing landscapes P = 0.06; Fig 3; median values provided in S1 Table, full results in S6 Table). The average movement by an avoider in changing landscapes ($\bar{x}=145.6\pm 25.62\text{m}$) was over twice as long as the average movement by an adapter/exploiter in these dynamic lands ($\bar{x}=70.4\pm 4.0\text{m}$). In part, this reflects the fact that reproductive failure in changing landscapes, which appears to stimulate movement (S3 Table), was most common among avoiders, (53.3% of 4132 adapter/exploiter territories followed over the course of study fledged young each year, but only 30.6% of 1943 avoider territories did so; X^2^_(1)_ = 274, P<0.0001). Across all landscapes, the average move by an avoider ($\bar{x}=112.96\pm 19.0\text{m}$, n = 41 birds) was only slightly greater than that by an adapter/exploiter ($\bar{x}=70.16\pm 4.5\text{m}$, n = 296 birds; P = 0.65; S6 Table).

Despite extended movements by avoiders living in changing landscapes relative to reserves or developments, actual land conversion within a bird’s territory was not closely associated with the distance moved. In changing landscapes, 33 of 263 territories experienced habitat transformation within 100m of their activity centers. On these territories, construction activities resulted in an average forest loss of 2968±638m^2^ (9.5% of the area) that was replaced by 1772±387m^2^ of built and 1196±533m^2^ of cleared, grass or shrub land. Birds that experienced local change in habitat composition did not move farther ($\bar{x}=64.4\pm 12.8\text{m}$, n = 33) than those birds that experienced no change ($\bar{x}=70.3\pm 5.4\text{m}$, n = 230; P = 0.30). Across all birds the distance moved was not correlated with the amount of change in forest, built, or other cover (all |*r*| <0.04, P > 0.55, n = 263). Forest loss within the year was not correlated with the distance moved by avoiders (r = 0.07, P = 0.76, n = 21). However, there was a tendency for Pacific wrens to move farthest from territories that had in prior years lost the most forest cover (r = 0.46, P_1-tailed_ = 0.07, n = 12). Distance moved by adapters/exploiters was not correlated with loss of forest or gain in anthropogenic lands (all |*r*| < 0.06, P > 0.33, n = 242).

### Does Movement Improve Reproduction or Enable Dispersers to Settle in Appropriate Habitat?

Movement within stable landscapes was more likely to improve an avoider’s reproductive success than was movement within changing landscapes. We had sufficient data to compare the probability of fledging offspring by individual avoiders the year preceding and the year after a move within changing (n = 12) and reserve (n = 10) lands. In changing landscapes only 2 (16.7%) avoiders that failed to fledge offspring and then moved succeeded in fledging offspring from their new territory. In contrast, 5 (50%) did so in reserved landscapes (Fisher’s exact test P_(1-tailed)_ = 0.11). In changing landscapes, 58% of avoiders were as or more likely to fledge young after moving to a new territory, but in reserves 90% maintained or improved their likelihood of fledging offspring (modeling the effect of landscape on maintaining the same or better versus worse fledging success after the move using a binomial regression: P = 0.12).

Regardless of landscape, adapters/exploiters that were or were not faithful to their previous breeding territory typically fledged young in consecutive years (94/152 pairs that remained within the average radius of their species’ territory fledged young in back-to-back years as did 81/155 that dispersed beyond the average species-specific territory boundary). However, following failure those that dispersed improved the likelihood of fledging a brood more often than did site faithful birds. Considering only birds that experienced different reproductive success in consecutive years, a majority (58.9%, n = 33) of dispersers were successful after the prior year’s failure. Only 41.1% (n = 23) of dispersers fledged offspring prior to the move and subsequently failed to do so on their new territory. In contrast, a minority of site faithful birds (37%, n = 17) were successful at fledging offspring the year following reproductive failure and many that were previously successful subsequently failed to fledge offspring (n = 29, 63%). Dispersers were more likely to succeed after failure and less likely to fail after success than were site faithful adapters/exploiters, regardless of landscape (Fisher’s Exact test: P = 0.03).

Movement within changing landscapes resulted in avoiders and adapters/exploiters acquiring territories of distinctive (and typical) land cover. After moving, the areas within a 100m radius of 22 avoider activity centers held on average 793±925m^2^ more forest land than did the abandoned territory, whereas the same areas for 261 adapters/exploiters held less forest cover and more built (231±172m^2^) and cleared (39±137m^2^) land. Both avoiders and adapters/exploiters ended up with mostly forest surrounding their activity centers, but avoiders had significantly more forest cover and by default less built and cleared land than did adapters (avoider: 24014±1084m^2^ forest, 2459±476m^2^ built, 4907±706m^2^ cleared; adapter/exploiter: 16909±299m^2^ forest, 6613±242m^2^ built, 7859±188m^2^ cleared; because of unit sum constraint we compare only forest: F_1, 281_ = 43.6, P < 0.0001).

**Fig 4:**
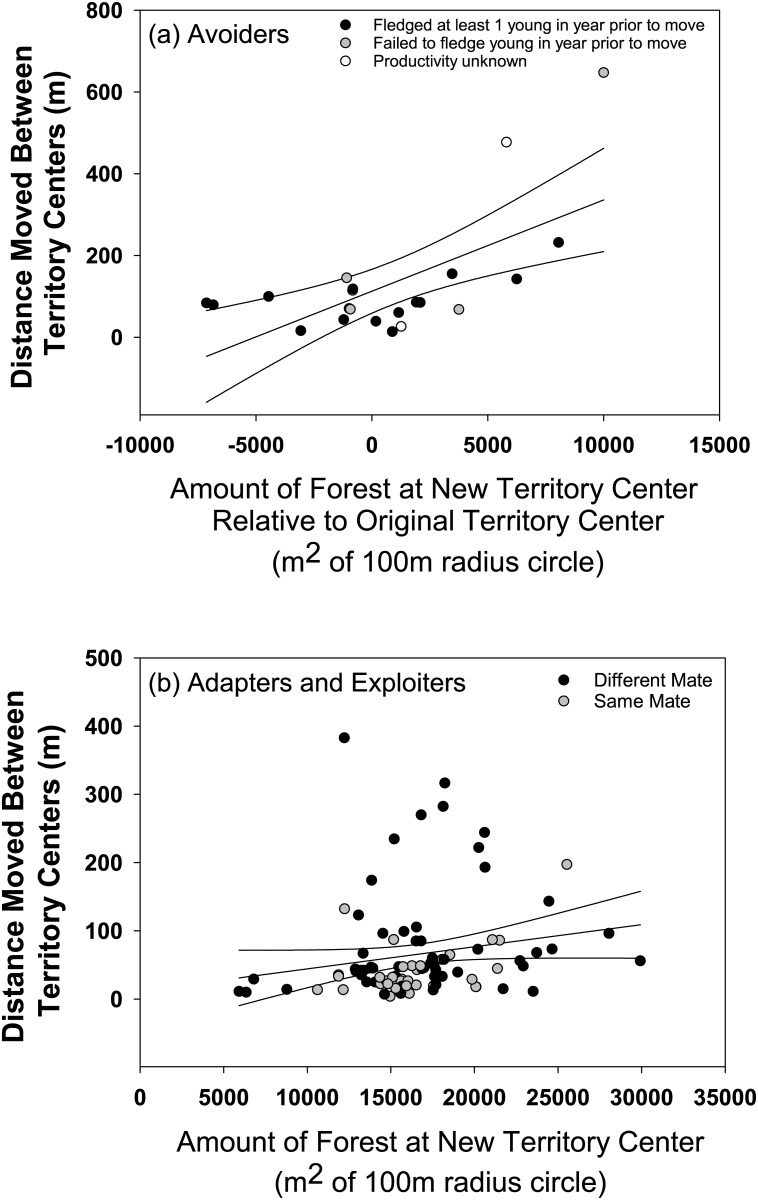
Breeding dispersal distance of adapters / exploiters (A) and avoiders (B) in response to vegetation changes associated with construction of new subdivisions. The most influential vegetation variable for each guild is used as the x-axis. For adapters / exploiters this was the total amount of forest within 100m of the new territory center. For avoiders it was the difference in total forest within 100m of the new territory center minus the old territory center (positive values indicate increases in forest cover at the territory each bird dispersed to. Shading of points indicates mate fidelity (A) or annual productivity (B), which were found to be important to each guild. Least-squares regression line ± 95% CI are fitted to data.

Moving to acquire a new territory in species’ typical vegetation appears of primary importance to avoiders, but not adapters/exploiters, in changing landscapes. We determined the annual movements in relation to productivity and resulting land cover for 19 avoiders. Those Pacific wrens and Swainson’s thrushes that moved farthest gained significantly greater forest cover but were equally likely to have fledged young the prior year as those that moved the least (Fig 4a; resulting forest: P = 0.002; prior fledging success: P = 0.79; full results in S7 Table). In contrast, the distance moved by 92 adapter/exploiters was more sensitive to whether a prior mate was retained than it was to the habitat acquired or a pair’s prior success at fledging offspring (Fig 4b; mate fidelity: P = 0.004, prior productivity: P = 0.90; resulting clearing: P = 0.33; note the results reported are for non-forested habitat which is appropriate for these species; full results in S8 Table).

## Discussion

### Change in Bird Communities During Urbanization

As native lands are urbanized animal communities rapidly shift from being dominated by sensitive, interior habitat specialists (‘avoiders;’ [12]) to those dominated by tolerant, flexible, edge and early successional species (‘adapters’ and ‘exploiters;’ [12]). Overall diversity in suburbs and exurbs may increase during this process [12, 14] and relative dominance attained by common species within the community may remain unchanged [22], but the composition of communities changes markedly [76–79]. By comparing reproduction, survivorship, and movements of sensitive ‘avoiders’ to those of tolerant ‘adapters’ and ‘exploiters’ we were able to demonstrate the demographic reasons why communities change in response to urbanization.

In the rapidly urbanizing Pacific Northwest of the USA, frequent reproductive failure of forest specialists such as the Pacific wren and Swainson’s thrush followed by their inability to obtain new breeding territories that enhance productivity cause population declines. The resident Pacific wren ultimately is excluded as a breeding species in most established subdivisions because of this reduced annual productivity and low adult survival (39%; [22]). In contrast, the migratory Swainson’s thrush may be sustained at low levels in established subdivisions despite low annual productivity because of higher adult survival (57%; [22]). As avoiders decline, adapters and exploiters increase to dominate suburban bird communities because of high breeding success and high annual survival during and after development (in most cases >50%; [22]). Moreover, when adapters and exploiters fail to reproduce in developments they disperse to new territories where their chances of successful reproduction are improved. Avoiders also practiced this adaptive strategy after less frequent reproductive failure in nearby forest reserves of moderate size (>75ha). There, the ability to disperse to higher quality territories, reproduce effectively, and survive enables Pacific wrens and Swainson’s thrushes to be two of the five most abundant species in northwestern forests [22].

### Hypotheses Concerning Variation in Breeding Dispersal and Mate Fidelity

Fidelity to territory in most birds is strong and therefore breeding dispersal is generally of short distance, frequently only a few territories away from the previous site [33]. This was true for all species studied in each landscape (S2 Table). If we consider movements shorter than the average territory dimension to be slight realignments of activity within the same area that an individual defended in the previous year, then site fidelity of species ranged from 16–64% (Table 2).

Variation within and among species in breeding dispersal allows us to appraise many of the hypotheses used to explain site fidelity in other species [33, 36]. Within all species, we found breeding dispersal to be closely mediated by prior reproductive success. As is often the case, dispersal distance was greater following annual failure to reproduce than it was following successful production of fledglings [44], and the dispersing individual did not consistently find greater success in their new territory [80]. Our results suggest that in optimal habitats dispersal following failure improves breeding productivity, but in suboptimal habitats this is not the case. Avoiders improved their breeding success by dispersing after failure in reserves, their optimal habitat (based on relative abundance and survival; [22]), but did not do so in suboptimal changing lands. In contrast, adapters and exploiters, which are abundant, productive and survive well in developed and undeveloped lands [22], improved their breeding success by dispersing after failure in these locations.

Low site fidelity in both migrant species (dark-eyed junco and Swainson’s thrush; Table 2) is consistent with hypotheses that earlier return to breeding sites by males than females increases breeding dispersal by females because their previous years’ mates have already paired with other females (bet-hedging hypothesis; [41] or because of active choice by females for the best available partner or territory (musical chairs hypothesis; [42]. Either alternative seems reasonable for Swainson’s thrushes because, although we resighted one third of previously banded males faithful to the prior year’s territory, we did not confirm any site fidelity for breeding females. The situation may be more complex for dark-eyed juncos because although their low site fidelity was associated with a high frequency of divorce, as expected by hypotheses linked to asynchronous returns, we did not observe greater dispersal by female juncos.

Land conversion due to urbanization results in juxtaposition of high and low quality territories and rapid changes in territory quality, which drive breeding dispersal in many species [34, 46]. We suggest this is a major reason for breeding dispersal by avoiders in changing lands. All Pacific wrens in changing lands lacked site fidelity, for example (S2 Table), in accordance with Stamp’s suggestion [48] that in fluctuating environments even high quality individuals may disperse. The influence of rapidly changing resource availability may also interact with the synchrony of returning to territory to affect dispersal in migrants regardless of their affinity for developed or reserved lands. Strong site fidelity by at least one sex of all species likely reflects the advantages of remaining on, or returning to, familiar ground with known nest sites, resources, and shelter [33].

Annual variation in density or availability of mates is known to cause variation in dispersal from year to year or from site to site [81, 82]. We did not assess this hypothesis directly, but dispersal distance within the three landscapes we studied was not related to a species’ abundance. Avoider abundance increases from developments to changing sites and reserves, while exploiter, and to a lesser extent adapter, abundance does the opposite [22]. We will assess the influence of density surrounding an individual pair at a finer scale in the future, but the current analysis suggests that immediate changes in landscape quality (avoiders) or pair compatibility (adapters/exploiters; Fig 4) rather than density per se motivates dispersal in dynamic lands.

### Increased Knowledge of Common Birds

Our long-term study of color-banded birds in a variety of landscapes increased our understanding of natural history in the urban ecosystem. Although established breeding pairs of songbirds move little, some individuals are capable of extensive forays across what we might consider inhospitable terrain; all species we studied moved several hundred meters across lands fully occupied by people. We confirmed findings from wilder locales concerning high site fidelity in Bewick’s wrens [53] and Pacific wrens [52]. However, in suburban settings relative to wildlands, we found divorce to be more common in song sparrows and dark-eyed juncos [49, 51] and site fidelity to be lower in Swainson’s thrushes and Pacific wrens [50, 52]. These decreases in stability of site ownership and pair bonding may reflect the dynamic nature of the suburban ecosystem. However, all developed settings may not be equal, as we found mate fidelity in spotted towhees to be even higher than reported for an urban population in nearby Portland, OR [54].

### Conservation Lessons From the Suburban Experiment

Increased human settlement of Earth challenges many species, but it also offers the urban ecologist an experimental arena within which to document causal connections between human action and ecological response. The changing landscapes we studied and compared to nearby established neighborhoods and forest reserves allowed us to better understand why avoiders decline and adapters and exploiters remain common in residential settings. Knowing that individual avoiders seek distant forests when the landscape they inhabit, and not just their immediate territory, is developed (Fig 4) suggests that the spatial extent of a neighborhood’s effect is greater than its immediate developed footprint. Moreover, the apparent inability of avoiders to improve their reproductive fitness by leaving changing lands suggests that the ecological consequences of developments are far-reaching and long lasting. (It is likely that some avoiders dispersed substantial distances and found success beyond the limited areas we searched.) Conservationists interested in sustaining sensitive species in urbanizing regions have a difficult task ahead of them, but our results emphasize the importance of nearby reserves where displaced avoiders might find a place to breed (and possibly adapt to a more urban life [15] and existing sensitive species might be able to continue to reproduce at sustainable levels and make adaptive changes in site and mate when reproduction fails. Conserving common species—the adapters and exploiters that live among us—is certainly easier than maintaining sensitive species. Our results suggest they are effective at breeding, surviving, and moving about in the places we also call home. Importantly, in the patches of forest, shrub, and garden that we studied, populations appear able to sequester significant human subsidies [31], while overcoming the fitness consequences of living near human structures [83, 84], predators [85], and invasive plants [20] in part because surviving individuals exercise adaptive options in site fidelity. The extent to which the Seattle area, which has only been settled by Europeans for 165 years and is surrounded by extensive wild areas, is unique will only be determined by studies such as ours in a greater variety of cities. Such comparative study is critically needed if we are to practice effective conservation in our increasingly urban world.

## Supporting Information

S1 Fig Average (+1SE) breeding dispersal distance by species within each of three landscapes. Sample size above error bars.
(EPS)

S1 Table Median dispersal distances within various subsets of the data. (DOCX)

S2 TableTerritory size (average radius in meters: ${\bar{x}}_{r}$) and site fidelity by songbirds in three landscapes.Site fidelity was assessed by calculating the number of moves between annual territory centers that exceeded in length the average radius in territories measured from 2002–2010. Species abbreviated as follows, Bewick’s wren: BEWR; dark-eyed junco: DEJU; song sparrow: SOSP; spotted towhee: SPTO; Swainson’s thrush: SWTH; Pacific wren: PAWR.(DOCX)

S3 Table Results of generalized linear mixed model with the dependent variable of annual distance moved between territory centers and the independent variables of landscape (Reserve, Developed, and Changing), Guild (binary), prior success at fledging young (binary), and the interaction between landscape and fledging success. Site was included as a random effect in the model. Fixed effects parameter estimates are shown (on the log-scale). Analysis conducted on 371 movements following known breeding outcomes by 13 Bewick’s wrens (3 fail), 25 dark-eyed juncos (5 fail), 185 song sparrows (39 fail), 118 spotted towhees (40 fail), 5 Swainson’s thrushes (5 fail), and 10 Pacific wrens (10 fail).
(DOCX)

S4 Table Results of generalized linear mixed model with the dependent variable of annual distance moved between territory centers by song sparrows, spotted towhees, and dark-eyed juncos, and the independent variables of landscape (Reserve, Developed, and Changing) and bond (broken due to death, broken due to divorce, intact). Site was included as a random effect in the model. Fixed effects parameter estimates are shown (on the log-scale). Analysis conducted on 148 movements following known pair bond status by 15 dark-eyed juncos (7 death, 6 divorce, 2 intact), 87 song sparrows (45 death 10 divorce, 32 intact), and 46 spotted towhees (26 death, 6 divorce, 14 intact).
(DOCX)

S5 Table Descriptive statistics of dispersal distances by male and female song sparrows, dark-eyed juncos, and spotted towhees following loss of mate to either divorce or apparent death versus retention of mate. (DOCX)

S6 Table Results of generalized linear mixed model with the dependent variable of average annual distance moved between territory centers and the independent variables of landscape (Reserve, Developed, and Changing), Guild (binary), and the interaction between landscape and Guild. Site was included as a random effect in the model. Fixed effects parameter estimates are shown (on the log-scale). Analysis conducted on 337 average movements by 9 Bewick’s wrens (2 changing, 2 developed, 5 reserve), 27 dark-eyed juncos (19 changing, 6 developed, 2 reserve), 162 song sparrows (90 changing, 51 developed, 21 reserve), 98 spotted towhees (63 changing, 14 developed, 21 reserve), 24 Pacific wrens (9 changing, 3 developed, 12 reserve), and 17 Swainson’s thrushes (9 changing, 2 developed, 6 reserve).
(DOCX)

S7 Table Results of generalized linear mixed model with the dependent variable of annual distance moved between territory centers in changing landscapes by avoiders and the independent variables of pixels of forest cover gained (standardized) and prior success at fledging young (binary). Site was included as a random effect in the model. Fixed effects parameter estimates are shown (on the log-scale). Analysis based on 19 movements (11 Pacific wren, 8 Swainson’s thrush).
(DOCX)

S8 Table Results of generalized linear mixed model with the dependent variable of annual distance moved between territory centers in changing landscapes by exploiters/adapters and the independent variables of pixels of non-forest cover gained (standardized), prior success at fledging young (binary), and mate retention (binary). Site was included as a random effect in the model. Fixed effects parameter estimates are shown (on the log-scale). Analysis based on 92 movements (1 Bewick’s wren, 9 dark-eyed juncos, 54 song sparrows, 28 spotted towhees).
(DOCX)

## References

[1] Angel S, Parent J, Civco DL, Blei A, Potere D. The dimensions of global urban expansion: estimates and projections for all countries, 2000–2050. Progress in Planning. 2011;75: 53–107.

[2] Shochat E, Warren PS, Faith SH, McIntyre NE, Hope D. From pattern to emerging processes in mechanistic urban ecology. Trends in Ecology and Evolution. 2006;21: 186–191. 10.1016/j.tree.2005.11.019 16701084

[3] Magle SB, Vernon VM, Crooks KR. Urban wildlife research: past, present, and future. Biological Conservation, 2012:155: 23–32.

[4] Kaye JP, Groffman PM, Grimm NB, Baker LA, Pouyat RV. A distinct urban biogeochemistry? Trends in Ecology and Evolution. 2006;21: 193–199. 10.1016/j.tree.2005.12.006 16701085

[5] Faeth SH, Warren PS, Shochat E, Marussich WA. Trophic dynamics in urban communities. BioScience. 2005;55: 399–407.

[6] Rodewald AD, Kearns LJ, Shustack DP. Anthropogenic resources subsidies decouple predator-prey relationships. Ecological Applications. 2011;21: 936–943. 21639056 10.1890/10-0863.1

[7] Fischer JD, Cleeton SH, Lyons TP, Miller JR. Urbanization and the predation paradox: the role of trophic dynamics in structuring vertebrate communities. BioScience. 2012;62: 809–818.

[8] Pickett STA, Cadenasso ML, Grove JM, Nilon CH, Pouyat RV, Zipperer WC, et al Urban ecological systems: linking terrestrial ecological, physical, and socioeconomic components of metropolitan areas. Annual Review of Ecology and Systematics. 2001;32: 127–157.

[9] Grimm NB, Grove JM, Pickett STA, Redman CL. Integrated approaches to long-term studies of urban ecological systems. BioScience. 2000;50: 571–584.

[10] Alberti M, Marzluff JM, Shulenberger E, Bradley G, Ryan C, ZumBrunnen C. Integrating humans into ecology: opportunities and challenges for studying urban ecosystems. BioScience. 2003;53: 1169–1179.

[11] Sattler T, Borcard D, Arlettaz R, Bontadina F, Legendre P, Obrist MK, et al Spider, bee and bird communities in cites are shaped by environmental control and high stochasticity. Ecology. 2010;91: 3343–3353. 21141195 10.1890/09-1810.1

[12] Blair RB. Land use and avian species diversity along an urban gradient. Ecological Applications. 1996;6: 506–519.

[13] Hansen AJ, Knight RL, Marzluff JM, Powell S, Brown K, Hernandez P, et al Effects of exurban development on biodiversity: patterns, mechanisms, research needs. Ecological Applications. 2005;15: 1893–1905.

[14] Marzluff JM. Island biogeography for an urbanizing world: how extinction and colonization may determine biological diversity in human-dominated landscapes. Urban Ecosystems. 2005;8:155–175.

[15] Marzluff JM. Urban evolutionary ecology. Studies in Avian Biology. 2012;45: 287–308.

[16] Natuhara Y, Hashimoto H. Spatial pattern and process in urban animal communities In: McDonnell MJ, Hahs AK, Greuste JH, editors. Ecology of Cities and Towns: A Comparative Approach. Cambridge: Cambridge University Press; 2009 pp 197–214.

[17] Rodewald AD, Shustack DP. Urban flight: understanding individual and populations-level responses of Nearctic-Neotropical migratory birds to urbanization. Journal of Animal Ecology. 2008;77: 833–91. 10.1111/j.1365-2656.2007.01313.x 17976185

[18] Sol D, Lapiedra O, González-Lagos C. Behavioural adjustments for a life in the city. Animal Behaviour. 2013 85: 1101–1112.

[19] Chamberlain DE, Cannon AR, Toms MP, Leech DI, Hatchwell BJ, Gaston KJ. Avian productivity in urban landscapes: a review and meta-analysis. Ibis. 2009;15: 1–18.

[20] Rodewald AD, Shustack DP, Hitchcock LE. Exotic shrubs as ephemeral ecological traps for nesting birds. Biological Invasions. 2010;12: 33–39.

[21] Ryder TB, Reitsma R, Evans B, Marra PP. Quantifying avian nest survival along an urbanization gradient using citizen and scientist generated data. Ecological Applications. 2010; 20: 419–426. 20405796 10.1890/09-0040.1

[22] Marzluff JM, Clucas B, Oleyar MD, DeLap J. The causal response of avian communities to suburban development: a quasi-experimental, longitudinal study. Urban Ecosystems. 2015;

[23] Evans BS, Ryder TB, Reitsma R, Hurlbert AH, Marra PP. Characterizing avian survival along a rural-to-urban land use gradient. Ecology. 2015;96: 1631–1640.

[24] Knight CR, Swaddle JP. Associations of anthropogenic activity and disturbance with fitness metrics of eastern bluebirds (*Sialia sialis*). Biological Conservation. 2007;138: 189–197.

[25] Whittaker KA, Marzluff JM. Species-specific survival and relative habitat use in an urban landscape during the postfledging period. Auk. 2009; 126: 1257–1276.

[26] Whittaker KA, Marzluff JM. Post-fledging mobility in an urban landscape. Studies in Avian Biology. 2012; 45: 183–198.

[27] Shochat E, Lerman SB, Anderies JM, Warren PS, Faeth SH, Nilon CH. Invasion, competition, and biodiversity loss in urban ecosystems. BioScience. 2010;60: 199–208.

[28] Kath J, Maron M, Dunn PK. Interspecific competition and small bird diversity in an urbanizing landscape. Landscape and Urban Planning. 2009;92:72–79.

[29] Farwell LS, Marzluff JM. A new bully on the block: Does urbanization promote Bewick’s wren aggressive exclusion of Pacific wrens? Biological Conservation. 2013;161: 128–141.

[30] Robb GN, McDonald RA, Chamberlain DE, Bearhop S. Food for thought: supplementary feeding as a driver of ecological change in avian populations. Frontiers in Ecology and the Environment. 2008;6: 476–484.

[31] Clucas B, Marzluff JM. Attitudes and actions toward birds in urban areas: human cultural differences influence bird behavior. Auk. 2012;129: 8–16.

[32] Fuller RA, Irvine KN, Davies ZG, Armsworth PR, Gaston KJ. Interactions between people and birds in urban landscapes. Studies in Avian Biology. 2012; 45: 249–266.

[33] Greenwood PJ, Harvey PH. The natal and breeding dispersal of birds. Annual Review of Ecology and Systematics. 1982;13: 1*–*21.

[34] Cline MH, Strong AM, Sillett TS, Rodenhouse NL, Holmes RT. Correlates and consequences of breeding dispersal in a migratory songbird. Auk. 2013; 130: 742–752.

[35] Dhondt AA. Changing mates. Trends in Ecology and Evolution. 2002;17: 55–56.

[36] Clobert J, Danchin E, Dhondt AA, Nichols JD, editors. Dispersal. Oxford: Oxford University Press; 2001.

[37] Breton AR, Nisbet ICT, Mostello CS, Hatch JJ. Age-dependent breeding dispersal and adult survival within a metapopulation of Comon Terns *Sterna hirundo*. Ibis. 2014;156: 534–547.

[38] Friedrich MJ, Hunt KL, Catlin DH, Fraser JD. The importance of site to mate choice: mate and site fidelity in Piping Plovers. Auk. 2015;132: 265–276.

[39] Ens BJ, Choudhury S, Black JM. Mate fidelity and divorce in monogamous birds In: Black JM, editor. Partnerships in birds. Oxford: Oxford University Press; 1996 pp. 344–395.

[40] Boggie MA, Mannan RW, Wissler C. Perennial pair bonds in an *Accipiter*: a behavioral response to an urbanized landscape? Journal of Raptor Research. 2015;49: 458–470.

[41] Handel CM, Gill RE. Mate fidelity and breeding site tenacity in a monogamous sandpiper, the Black Turnstone. Animal Behaviour. 2000;60: 471–481. 10.1006/anbe.2000.1505 11032650

[42] Dhondt AA, Adriaensen F. Causes and effects of divorce in the Blue Tit *Parus caeruleus*. Journal of Animal Ecology. 1994;63: 979–987.

[43] Davies NB. Sexual conflict and the polygamy threshold. Animal Behaviour. 1989;38: 226–234.

[44] Dubois F, Cézilly F. Breeding success and mate retention in birds: A meta-analysis. Behavioral Ecology and Sociobiology. 2002;52: 357–364.

[45] Newton I, Wyllie I. Monogamy in the Sparrowhawk In: In: Black JM, editor. Partnerships in birds. Oxford: Oxford University Press; 1996 pp. 249–267.

[46] Gutiérrez RJ, Lahaye WS, Zimmerman GS. Breeding dispersal in an isolated population of Spotted Owls *Strix occidentalis*: evidence for improved reproductive output. Ibis. 2011;153: 592–600.

[47] Terraube J, Vaskod V, Korpimäki E. Mechanisms and reproductive consequences of breeding dispersal in a specialist predator under temporally varying food conditions. Oikos. 2015;124: 762–771.

[48] Stamps JA (2001) Habitat selection by dispersers: integrating proximate and ultimate approaches In: Clobert J, Danchin E, Dhondt AA, Nichols JD, editors. Dispersal. Oxford: Oxford University Press; 2001. pp. 230–242.

[49] Arcese P, Sogge MK, Marr AB, Patten MA. Song Sparrow (*Melospiza melodia*) In: Poole A, editor. The Birds of North America Online.Ithaca: Cornell Lab of Ornithology; 2002.

[50] Mack DE, Yong W. Swainson's Thrush (*Catharus ustulatus*) In: Poole A, editor. The Birds of North America Online. Ithaca: Cornell Lab of Ornithology; 2000.

[51] Nolan V Jr, Ketterson ED, Cristol DA, Rogers CM, Clotfelter ED, Titus RC, et al Dark-eyed Junco (*Junco hyemalis*) In: Poole A, editor. The Birds of North America Online. Ithaca: Cornell Lab of Ornithology; 2002.

[52] Toews DPL, Irwin DE. Pacific Wren (*Troglodytes pacificus*) In: Poole A, editor. The Birds of North America Online. Ithaca: Cornell Lab of Ornithology; 2012.

[53] Kennedy ED, White DW. Bewick's Wren (*Thryomanes bewickii*) In: Poole A, editor. The Birds of North America Online. Ithaca: Cornell Lab of Ornithology; 2013.

[54] Bartos Smith S, Greenlaw JS. Spotted Towhee (*Pipilo maculatus*) In: Poole A, editor. The Birds of North America Online. Ithaca: Cornell Lab of Ornithology; 2015.

[55] Rodewald AD, Gehrt SD. Wildlife population dynamics in urban landscapes In: McCleery RA, Moorman CE, Peterson MN, editors. Urban Wildlife Conservation: Theory and Practice. New York: Springer; 2014 pp. 117–147.

[56] Donnelly RE Marzluff JM. Relative importance of habitat quantity, structure, and spatial pattern to birds in urbanizing environments. Urban Ecosystems. 2006;9: 99–117.

[57] Robinson L, Newell JP, Marzluff JM. Twenty-five years of sprawl in the Seattle region: growth management responses and implications for conservation. Landscape and Urban Planning. 2005;71: 51–72.

[58] Hepinstall JA, Alberti M, Marzluff JM. Predicting land cover change and avian community responses in rapidly urbanizing environments. Landscape Ecology. 2008;23: 1257–1276.

[59] Donnelly R, Marzluff JM. Importance of reserve size and landscape context to urban bird conservation. Conservation Biology. 2004;18: 733–745.

[60] Blewett CM, Marzluff JM. Effects of urban sprawl on snags and the abundance and productivity of cavity-nesting birds. Condor. 2005;107: 677–692.

[61] Pyle P. Identification Guide to North American Birds, Part 1. Bolinas, CA: Slate Creek Press; 1997.

[62] International Bird Census Committee. An international standard for a mapping method in bird census work recommended by the International Bird Census Committee. Audubon Field Notes. 1970;24: 722–726.

[63] Vickery PD, Hunter ML Jr, Wells JV. Use of a new reproductive index to evaluate relationship between habitat quality and breeding success. Auk. 1992;109: 697–705.

[64] Ralph CJ, Geupel GR, Pyle P, Martin TE, Desante DF. Handbook of Field Methods for Monitoring Landbirds General Technical Report PSW-GTR-144. Albany, CA: U.S. Department of Agriculture, Forest Service, Pacific Southwest Research Station; 1993.

[65] Christoferson LL, Morrison ML. Integrating methods to determine breeding and nesting status of three western songbirds. Wildlife Society Bulletin. 2001;29: 688–696.

[66] ESRI. ArcGIS Desktop: Release 10. Redlands, CA: Environmental Systems Research Institute; 2011.

[67] Halabisky M, Moskal LM, Hall SA. Object-based classification of semi-arid wetlands. Journal of Applied Remote Sensing. 2011;5:13.

[68] Camara G, Cartaxo R, Souza M, Freitas UM, Garrido J. Spring: integrating remote sensing and GIS by object-oriented data modelling. Computers and Graphics. 1996;20: 395–404.

[69] Foody GM. Status of land cover classification accuracy assessment. Remote Sensing of Environment. 2002;80: 185–201.

[70] Van Coillie FMB, Gardin S, Anseel F, Duyck W, Verbeke LPC, De Wulf RR. Variability of operator performance in remote-sensing image interpretation: the importance of human and external factors. International Journal of Remote Sensing. 2014;35: 754–778.

[71] IBM Corporation. IBM SPSS Statistics. V. 19 Armonk: New York; 2010.

[72] R Core Team. R: A Language and Environment for Statistical Computing. Vienna, Austria: R Foundation for Statistical Computing; 2016.

[73] Fisher RA. Theory of statistical estimation. Proceedings of the Cambridge Philosophical Society. 1925;22: 700–725.

[74] Robinson DH, Wainer H. On the past and future of null hypothesis significance testing. Journal of Wildlife Management. 2002;66: 263–271.

[75] Armstrong RA. When to use the Bonferroni correction. Ophthalmic and Physiological Optics. 2014;34: 502–508. 10.1111/opo.12131 24697967

[76] Aldrich JW, Coffin RW. Breeding bird populations from forest to suburbia after thirty-seven years. American Birds. 1980;34: 3–7.

[77] Cramp S. Changes in the breeding birds of inner London since 1900. Proceedings of the International Ornithological Congress. 1980;17: 1316–1320.

[78] Reecher HF, Serventy DL. Long term changes in the relative abundances of birds in Kings Park, Perth, Western Australia. Conservation Biology. 1991;5: 90–102.

[79] Gehlbach FR. Native Texas avifauna altered by suburban entrapment and method for easing assessing natural avifaunal value. Bulletin of the Texas Ornithological Society. 2005;38: 35–47.

[80] Włodarczyk R, Weiloch M, Czyż S, Dodlata PT, Minias P. Natal and breeding dispersal in Mute Swans *Cygnus olor*: influence of sex, mate switching and reproductive success. Acta Ornithologica. 2013;48: 237–244.

[81] Marzluff JM, Balda RP. Social, demographic, and evolutionary consequences of dispersal in a group-living bird, the pinyon jay. Ecology. 1989;70: 316–328.

[82] Liebgold EB, Gerlach NM, Ketterson ED. Similarity in temporal variation in sex-biased dispersal over short and long distances in the dark-eyed junco, *Junco hyemalis*. Molecular Ecology. 2013;22: 5548–5560. 24730036 10.1111/mec.12508

[83] Loss SR, Will T, Loss SS, Marra PP. Bird-building collisions in the United States: estimates of annual mortality and species vulnerability. Condor. 2014;116: 8–23.

[84] Longcore T, Rich C, Mineau P, MacDonald B, Bert DG, Sullivan LM, et al An estimate of avian mortality at communication towers in the United States and Canada. PLoS One. 2012;7, e34025 10.1371/journal.pone.0034025 22558082 PMC3338802

[85] Loss SR, Will T, Marra PP. The impact of free-ranging domestic cats on wildlife in the United States. Nature Communications. 2013;4: 1396 10.1038/ncomms2380 23360987

