# Supplementary material for: Breeding Dispersal by Birds in a Dynamic Urban Ecosystem
Source: PLoS One. 2016 Dec 28;11(12):e0167829. doi: 10.1371/journal.pone.0167829 (PMC5193330; doi:10.1371/journal.pone.0167829)
Supplement: S1 Table — (DOCX) [file pone.0167829.s002.docx]

**S1 Table. Median dispersal distances within various subsets of the data**.

| Subset | N | Median  (m) |
| --- | --- | --- |
| Avoiders in Reserved Landscapes | 18 | 76.0 |
| Avoiders in Developed Landscapes | 5 | 51.0 |
| Avoiders in Changing Landscapes | 18 | 84.6 |
| Exploiters/Adapters in Reserved Landscapes | 49 | 45.5 |
| Exploiters/Adapters in Developed Landscapes | 72 | 36.4 |
| Exploiters/Adapters in Changing Landscapes | 172 | 44.6 |
| Failed Breeders in All Landscapes | 102 | 48.1 |
| Successful Breeders in All Landscapes | 269 | 42.8 |
| Failed Breeders in Changing Landscapes | 52 | 42.4 |
| Divorcees, Exploiters/Adapters in all Landscapes | 23 | 60.4 |
| Widows and Widowers, Exploiters/Adapters in all Landscapes | 77 | 43.9 |
| Birds Retaining Mate, Exploiters/Adapters in all Landscapes | 48 | 29.1 |
