# Supplementary material for: Breeding Dispersal by Birds in a Dynamic Urban Ecosystem
Source: PLoS One. 2016 Dec 28;11(12):e0167829. doi: 10.1371/journal.pone.0167829 (PMC5193330; doi:10.1371/journal.pone.0167829)
Supplement: S2 Table — Site fidelity was assessed by calculating the number of moves between annual territory centers that exceeded in length the average radius in territories measured from 2002–2010. Species abbreviated as follows, Bewick’s wren: BEWR; dark-eyed junco: DEJU; song sparrow: SOSP; spotted towhee: SPTO; Swainson’s thrush: SWTH; Pacific wren: PAWR. (DOCX) [file pone.0167829.s003.docx]

**S2 Table. Territory size (average radius in meters:**${\bar{\boldsymbol{x}}}_{\boldsymbol{r}}$**) and site fidelity by songbirds in three landscapes. Site fidelity was assessed by calculating the number of moves between annual territory centers that exceeded in length the average radius in territories measured from 2002-2010. Species abbreviated as follows, Bewick’s wren: BEWR; dark-eyed junco: DEJU; song sparrow: SOSP; spotted towhee: SPTO; Swainson’s thrush: SWTH; Pacific wren: PAWR.**

|  | Changing Landscape | | Developed Landscape | | Reserved Forest Landscape | | |  |
| --- | --- | --- | --- | --- | --- | --- | --- | --- |
|  |  | |  | |  | | |  |
| Guild  SPECIES | $\bar{x}_{r}$  (n, SE) | Site Fidelity  % moves < $\bar{x}_{r}$ (n) | $\bar{x}_{r}$  (n, SE) | Site Fidelity  % moves < $\bar{x}_{r}$ (n) | | $\bar{x}_{r}$  (n, SE) | Site Fidelity  % moves < $\bar{x}_{r}$ (n) | |
| Adapter/Exploiter |  |  |  |  | |  |  | |
| BEWR | 45.2  (91, 1.3) | 66.7  (6) | 53.8  (33, 2.1) | 0.0  (2) | | 39.7  (27, 1.6) | 83.3  (6) | |
| DEJU | 42.4  (100, 2.0) | 9.5  (21) | 35.3  (44, 1.9) | 28.6  (7) | | 46.6  (61, 3.1) | 33.3  (3) | |
| SOSP | 37.1  (181, 1.0) | 47.7  (151) | 36.5  (111, 1.4) | 53.3  (76) | | 47.8  (77, 2.5) | 46.7  (30) | |
| SPTO | 44.8  (173, 1.4) | 49.0  (96) | 40.1  (86, 1.6) | 52.6  (19) | | 46.7  (101, 2.5) | 57.5  (40) | |
| Avoider |  |  |  |  | |  |  | |
| PAWR | 35.1  (119, 0.85) | 0.0  (10) | 38.7  (52, 1.5) | 66.7  (3) | | 37.5  (97, 1.7) | 21.4  (14) | |
| SWTH | 49.5  (105, 1.4) | 36.4  (11) | 45.7  (53, 2.2) | 0.0  (2) | | 52.7  (68, 2.5) | 42.9  (7) | |
