# Supplementary material for: Breeding Dispersal by Birds in a Dynamic Urban Ecosystem
Source: PLoS One. 2016 Dec 28;11(12):e0167829. doi: 10.1371/journal.pone.0167829 (PMC5193330; doi:10.1371/journal.pone.0167829)
Supplement: S3 Table — Site was included as a random effect in the model. Fixed effects parameter estimates are shown (on the log-scale). Analysis conducted on 371 movements following known breeding outcomes by 13 Bewick’s wrens (3 fail), 25 dark-eyed juncos (5 fail), 185 song sparrows (39 fail), 118 spotted towhees (40 fail), 5 Swainson’s thrushes (5 fail), and 10 Pacific wrens (10 fail). (DOCX) [file pone.0167829.s004.docx]

**S3 Table. Results of generalized linear mixed model with the dependent variable of annual distance moved between territory centers and the independent variables of landscape (Reserve, Developed, and Changing), Guild (binary), prior success at fledging young (binary), and the interaction between landscape and fledging success. Site was included as a random effect in the model. Fixed effects parameter estimates are shown (on the log-scale). Analysis conducted on 371 movements following known breeding outcomes by 13 Bewick’s wrens (3 fail), 25 dark-eyed juncos (5 fail), 185 song sparrows (39 fail), 118 spotted towhees (40 fail), 5 Swainson’s thrushes (5 fail), and 10 Pacific wrens (10 fail).**

|  | Estimate | Std. Error | t value | p-value |
| --- | --- | --- | --- | --- |
| Intercept | 4.14 | 0.20 | 20.80 | <0.001 |
| Developed | -0.07 | 0.30 | -0.23 | 0.82 |
| Changing | 0.13 | 0.25 | 0.54 | 0.59 |
| Fledge Success | 0.28 | 0.22 | 1.26 | 0.21 |
| Guild (Avoider) | 0.43 | 0.19 | 2.27 | 0.02 |
| Developed:FledgeSuccess | -0.41 | 0.33 | -1.24 | 0.21 |
| Changing:FledgeSuccess | -0.54 | 0.26 | -2.07 | 0.04 |
