# Supplementary material for: Breeding Dispersal by Birds in a Dynamic Urban Ecosystem
Source: PLoS One. 2016 Dec 28;11(12):e0167829. doi: 10.1371/journal.pone.0167829 (PMC5193330; doi:10.1371/journal.pone.0167829)
Supplement: S4 Table — Site was included as a random effect in the model. Fixed effects parameter estimates are shown (on the log-scale). Analysis conducted on 148 movements following known pair bond status by 15 dark-eyed juncos (7 death, 6 divorce, 2 intact), 87 song sparrows (45 death 10 divorce, 32 intact), and 46 spotted towhees (26 death, 6 divorce, 14 intact). (DOCX) [file pone.0167829.s005.docx]

**S4 Table. Results of generalized linear mixed model with the dependent variable of annual distance moved between territory centers by song sparrows, spotted towhees, and dark-eyed juncos, and the independent variables of landscape (Reserve, Developed, and Changing) and bond (broken due to death, broken due to divorce, intact). Site was included as a random effect in the model. Fixed effects parameter estimates are shown (on the log-scale). Analysis conducted on 148 movements following known pair bond status by 15 dark-eyed juncos (7 death, 6 divorce, 2 intact), 87 song sparrows (45 death 10 divorce, 32 intact), and 46 spotted towhees (26 death, 6 divorce, 14 intact).**

|  | Estimate | Std. Error | t value | p-value |
| --- | --- | --- | --- | --- |
| Intercept | 4.45 | 0.41 | 10.74 | <0.001 |
| Developed | -0.72 | 0.49 | -1.45 | 0.15 |
| Changing | -0.32 | 0.45 | -0.71 | 0.48 |
| Divorce | 0.89 | 0.23 | 3.83 | <0.001 |
| Intact | -0.23 | 0.18 | -1.28 | 0.20 |
