# Supplementary material for: Breeding Dispersal by Birds in a Dynamic Urban Ecosystem
Source: PLoS One. 2016 Dec 28;11(12):e0167829. doi: 10.1371/journal.pone.0167829 (PMC5193330; doi:10.1371/journal.pone.0167829)
Supplement: S5 Table — (DOCX) [file pone.0167829.s006.docx]

**S5 Table. Descriptive statistics of dispersal distances by male and female song sparrows, dark-eyed juncos, and spotted towhees following loss of mate to either divorce or apparent death versus retention of mate.**

| Category | N | Mean (m) | SE (m) | Median (m) |
| --- | --- | --- | --- | --- |
| Female: Mate dead | 14 | 84.2 | 27.7 | 42.8 |
| Female: Mate divorced | 12 | 139.3 | 34.2 | 70.5 |
| Female: Mate retained | 22 | 67.2 | 21.4 | 30.4 |
| Male: Mate dead | 63 | 54.7 | 6.6 | 43.9 |
| Male: Mate divorced | 11 | 127.4 | 45.2 | 60.4 |
| Male: Mate retained | 26 | 55.1 | 17.1 | 29.1 |
