# Supplementary material for: Breeding Dispersal by Birds in a Dynamic Urban Ecosystem
Source: PLoS One. 2016 Dec 28;11(12):e0167829. doi: 10.1371/journal.pone.0167829 (PMC5193330; doi:10.1371/journal.pone.0167829)
Supplement: S6 Table — Site was included as a random effect in the model. Fixed effects parameter estimates are shown (on the log-scale). Analysis conducted on 337 average movements by 9 Bewick’s wrens (2 changing, 2 developed, 5 reserve), 27 dark-eyed juncos (19 changing, 6 developed, 2 reserve), 162 song sparrows (90 changing, 51 developed, 21 reserve), 98 spotted towhees (63 changing, 14 developed, 21 reserve), 24 Pacific wrens (9 changing, 3 developed, 12 reserve), and 17 Swainson’s thrushes (9 changing, 2 developed, 6 reserve). (DOCX) [file pone.0167829.s007.docx]

**S6 Table. Results of generalized linear mixed model with the dependent variable of average annual distance moved between territory centers and the independent variables of landscape (Reserve, Developed, and Changing), Guild (binary), and the interaction between landscape and Guild. Site was included as a random effect in the model. Fixed effects parameter estimates are shown (on the log-scale). Analysis conducted on 337 average movements by 9 Bewick’s wrens (2 changing, 2 developed, 5 reserve), 27 dark-eyed juncos (19 changing, 6 developed, 2 reserve), 162 song sparrows (90 changing, 51 developed, 21 reserve), 98 spotted towhees (63 changing, 14 developed, 21 reserve), 24 Pacific wrens (9 changing, 3 developed, 12 reserve), and 17 Swainson’s thrushes (9 changing, 2 developed, 6 reserve).**

|  | Estimate | St. Error | t-value | P-value |
| --- | --- | --- | --- | --- |
| Intercept | 4.47 | 0.16 | 27.18 | <0.001 |
| Changing | -0.26 | 0.19 | -1.37 | 0.17 |
| Developed | -0.41 | 0.21 | -1.95 | 0.05 |
| Guild (Avoider) | 0.11 | 0.24 | 0.46 | 0.65 |
| Changing:Avoider | 0.62 | 0.33 | 1.91 | 0.06 |
| Developed:Avoider | -0.20 | 0.47 | -0.43 | 0.67 |
