# Supplementary material for: Breeding Dispersal by Birds in a Dynamic Urban Ecosystem
Source: PLoS One. 2016 Dec 28;11(12):e0167829. doi: 10.1371/journal.pone.0167829 (PMC5193330; doi:10.1371/journal.pone.0167829)
Supplement: S7 Table — Site was included as a random effect in the model. Fixed effects parameter estimates are shown (on the log-scale). Analysis based on 19 movements (11 Pacific wren, 8 Swainson’s thrush). (DOCX) [file pone.0167829.s008.docx]

**S7 Table. Results of generalized linear mixed model with the dependent variable of annual distance moved between territory centers in changing landscapes by avoiders and the independent variables of pixels of forest cover gained (standardized) and prior success at fledging young (binary). Site was included as a random effect in the model. Fixed effects parameter estimates are shown (on the log-scale). Analysis based on 19 movements (11 Pacific wren, 8 Swainson’s thrush).**

|  | Estimate | Std. Error | t value | p-value |
| --- | --- | --- | --- | --- |
| Intercept | 4.67 | 0.32 | 14.44 | <0.001 |
| Fledge Success | -0.09 | 0.34 | -0.27 | 0.79 |
| Forest Cover | 0.43 | 0.14 | 3.16 | 0.002 |
