# Supplementary material for: Breeding Dispersal by Birds in a Dynamic Urban Ecosystem
Source: PLoS One. 2016 Dec 28;11(12):e0167829. doi: 10.1371/journal.pone.0167829 (PMC5193330; doi:10.1371/journal.pone.0167829)
Supplement: S8 Table — Site was included as a random effect in the model. Fixed effects parameter estimates are shown (on the log-scale). Analysis based on 92 movements (1 Bewick’s wren, 9 dark-eyed juncos, 54 song sparrows, 28 spotted towhees). (DOCX) [file pone.0167829.s009.docx]

**S8 Table. Results of generalized linear mixed model with the dependent variable of annual distance moved between territory centers in changing landscapes by exploiters/adapters and the independent variables of pixels of non-forest cover gained (standardized), prior success at fledging young (binary), and mate retention (binary). Site was included as a random effect in the model. Fixed effects parameter estimates are shown (on the log-scale). Analysis based on 92 movements (1 Bewick’s wren, 9 dark-eyed juncos, 54 song sparrows, 28 spotted towhees).**

|  | Estimate | Std. Error | t value | p-value |
| --- | --- | --- | --- | --- |
| Intercept | 4.30 | 0.28 | 15.30 | <0.001 |
| Fledge Success | -0.03 | 0.27 | -0.12 | 0.90 |
| Mate Retention = same | -0.57 | 0.20 | -2.86 | 0.004 |
| Non-forest Cover | -0.09 | 0.09 | -0.96 | 0.34 |
